# Supplementary material for: Comparative Mitogenomics Analysis Revealed Evolutionary Divergence among Neopestalotiopsis Species Complex (Fungi: Xylariales)
Source: Int J Mol Sci. 2024 Mar 7;25(6):3093. doi: 10.3390/ijms25063093 (PMC10970013; doi:10.3390/ijms25063093)
Supplement: Supplementary file 1 [file ijms-25-03093-s001.zip › ijms-2851241-supplementary.pdf]

Supplementary Materials

Supplementary Table S1: Strain classification and Mitogenome features

Items	*Neopestalotiopsis cubana* WZS16	*Neopestalotiopsis cubana* LD08	*Neopestalotiopsis cubana* BT03	*Pestalotiopsis fici* W106-1
Phylum	Ascomycota	Ascomycota	Ascomycota	Ascomycota
Order	*Xylariales*	*Xylariales*	*Xylariales*	*Xylariales*
Family	*Sporocadaceae*	*Sporocadaceae*	*Sporocadaceae*	*Sporocadaceae*
GenBank accession number	NC_071220.1	OQ707026.1	OQ707025.1	KX870077.1
Genome size（bp）	38,666	33,846	32,593	69,529
GC content（%）	27.48	28.20	28.26	28.45
AT-skew	-0.026	-0.035	-0.040	0.007
GC-skew	0.117	0.118	0.118	0.107
No of ORFs	10	3	3	9
No of introns	0	1	0	18
No of tRNAs	34	31	31	32

Supplementary Table S2: Start and stop codons of core protein coding genes

**Species**	***atp6***		***atp8***		***atp9***		***cob***		***cox1***	
	start	stop	start	stop	start	stop	start	stop	start	stop
N.cubana WZS16	ATG	TAA	ATG	TAA	ATG	TAA	ATG	TAA	ATG	TAA
N.cubana LD08	ATG	TAA	ATG	TAA	ATG	TAA	ATG	TAA	ATG	TAA
N.cubana BT03	ATG	TAA	ATG	TAA	ATG	TAA	ATG	TAA	ATG	TAA
P.fici W106-1	ATG	TAA	ATG	TAA	ATG	TAA	ATG	TAG	ATG	TAA

cox2		***cox3***		***nad1***		***nad2***		***nad3***	
start	stop	start	stop	start	stop	start	stop	start	stop
ATG	TAA	ATG	TAA	ATG	TAA	ATG	TAA	ATG	TAA
ATG	TAA	ATG	TAA	ATG	TAA	ATG	TAA	ATG	TAA
ATG	TAA	ATG	TAA	ATG	TAA	ATG	TAA	ATG	TAA
ATG	TAA	ATG	TAA	ATG	TAA	ATG	TAA	ATG	TAA

nad4		***nad4L***		***nad5***		***nad6***		***rps3***	
start	stop	start	stop	start	stop	start	stop	start	stop
ATG	TAG	ATG	TAA	ATG	TAG	ATG	TAA	ATG	TAA
ATG	TAG	ATG	TAA	ATG	TAG	ATG	TAA	ATG	TAA
ATG	TAG	ATG	TAA	ATG	TAG	ATG	TAA	ATG	TAA
ATG	TAG	ATG	TAA	ATG	TAA	ATG	TAA	ATG	TAA

Supplementary Table S3: Information of tRNAs in the mitochondrial genome

N.cubana WSZ16	*N.cubana BT03*	*N.cubana LD08*	*P.ficiw106-1*
trnT(tgt)	*trnT(tgt)*	*trnT(tgt)*	*trnT(tgt)*
trnE(ttc)	*trnE(ttc)*	*trnE(ttc)*	*trnE(ttc)*
trnM(cat)	*trnM(cat)*	*trnM(cat)*	*trnM(cat)*
trnM(cat)	*trnM(cat)*	*trnM(cat)*	*trnM(cat)*
trnL(taa)	*trnL(taa)*	*trnL(taa)*	*trnL(taa)*
trnA(tgc)	*trnA(tgc)*	*trnA(tgc)*	*trnA(tgc)*
trnF(gaa)	*trnF(gaa)*	*trnF(gaa)*	*trnF(gaa)*
trnL(tag)	*trnL(tag)*	*trnL(tag)*	*trnL(tag)*
trnQ(ttg)	*trnQ(ttg)*	*trnQ(ttg)*	*trnQ(ttg)*
trnH(gtg)	*trnH(gtg)*	*trnH(gtg)*	*trnH(gtg)*
trnM(cat)	*trnM(cat)*	*trnM(cat)*	*trnM(cat)*
			trnY(ata)
trnV(tac)	*trnV(tac)*	*trnV(tac)*	
trnR(tct)	*trnR(tct)*	*trnR(tct)*	*trnR(tct)*
trnC(gca)	*trnC(gca)*	*trnC(gca)*	*trnC(gca)*
			trnC(gca)
trnR(acg)	*trnR(acg)*	*trnR(acg)*	*trnR(acg)*
trnR(tct)	*trnR(tct)*	*trnR(tct)*	*trnR(tct)*
trnR(tct)	*trnR(tct)*	*trnR(tct)*	*trnR(tct)*
trnY(gta)	*trnY(gta)*	*trnY(gta)*	*trnY(gta)*
trnN(gtt)	*trnN(gtt)*	*trnN(gtt)*	*trnN(gtt)*
trnK(ttt)	*trnK(ttt)*	*trnK(ttt)*	*trnK(ttt)*
trnG(acc)			*trnG(acc)*
trnG(tcc)	*trnG(tcc)*	*trnG(tcc)*	
trnL(caa)			
trnG(tcc)			
trnD(gtc)	*trnD(gtc)*	*trnD(gtc)*	*trnD(gtc)*
trnS(gct)	*trnS(gct)*	*trnS(gct)*	*trnS(gct)*
trnW(tca)	*trnW(tca)*	*trnW(tca)*	*trnW(tca)*
trnP(tgg)	*trnP(tgg)*	*trnP(tgg)*	*trnP(tgg)*
trnS(tga)	*trnS(tga)*	*trnS(tga)*	*trnS(tga)*
trnD(gtc)	*trnD(gtc)*	*trnD(gtc)*	*trnD(gtc)*
trnV(tac)	*trnV(tac)*	*trnV(tac)*	*trnV(tac)*
trnV(tac)	*trnV(tac)*	*trnV(tac)*	*trnV(tac)*
trnI(gat)	*trnI(gat)*	*trnI(gat)*	*trnI(gat)*
trnP(tgg)	*trnP(tgg)*	*trnP(tgg)*	*trnP(tgg)*

Red font indicates tRNAs common to the four species

Supplementary Table S4: Repetitive sequences positions in the mitogenome of *N. cubana* LD08 revealed by REPuter

Size	POS1 start	POS1 end	Type	Size	POS2 start	POS2 end
22	3004	3026	F	22	23431	23453
22	13744	13766	F	22	15361	15383
22	13744	13766	F	22	17227	17249
22	13744	13766	F	22	30847	30869
22	1600	1622	F	22	13223	13245
22	3754	3776	F	22	33160	33182
22	5096	5118	F	22	9885	9907
22	14915	14937	F	22	32759	32781
22	15929	15951	F	22	33142	33164
22	33	55	F	22	6805	6827
22	906	928	F	22	31062	31084
22	1115	1137	F	22	33349	33371
22	1250	1272	F	22	19434	19456
22	1722	1744	F	22	23865	23887
22	1771	1793	F	22	14948	14970
22	2112	2134	F	22	5770	5792
22	3109	3131	F	22	27251	27273
22	3118	3140	F	22	23416	23438
22	3206	3228	F	22	31345	31367
22	3422	3444	F	22	5751	5773
22	3490	3512	F	22	15875	15897
22	3494	3516	F	22	16002	16024
22	4576	4598	F	22	27911	27933
22	4832	4854	F	22	29820	29842
22	5007	5029	F	22	9853	9875
22	5057	5079	F	22	12942	12964
22	5095	5117	F	22	23715	23737
22	5174	5196	F	22	29691	29713
22	5181	5203	F	22	25725	25747
22	5427	5449	F	22	8946	8968
22	5666	5688	F	22	32044	32066
22	5681	5703	F	22	19300	19322
22	5756	5778	F	22	7681	7703
22	5958	5980	F	22	17602	17624
22	6315	6337	F	22	22860	22882
22	6325	6347	F	22	12408	12430
22	6606	6628	F	22	28663	28685
22	6851	6873	F	22	33598	33620
22	6911	6933	F	22	14699	14721
22	7009	7031	F	22	32617	32639

22	7436	7458	F	22	18061	18083
22	8429	8451	F	22	32065	32087
22	8856	8878	F	22	32066	32088
22	9651	9673	F	22	13093	13115
22	9654	9676	F	22	15980	16002
22	9681	9703	F	22	14803	14825
22	11541	11563	F	22	22876	22898
22	13096	13118	F	22	26950	26972
22	14098	14120	F	22	15911	15933
22	14801	14823	F	22	26945	26967
22	15911	15933	F	22	19529	19551
22	18325	18347	F	22	18337	18359
22	19816	19838	F	22	23371	23393
22	22525	22547	F	22	27028	27050
22	23311	23333	F	22	23317	23339
22	25726	25748	F	22	33803	33825
22	27077	27099	F	22	31252	31274
22	27085	27107	F	22	33174	33196
22	29561	29583	F	22	29811	29833
22	30984	31006	F	22	33338	33360
22	31881	31903	F	22	32067	32089
23	5184	5207	F	23	29162	29185
23	9918	9941	F	23	12865	12888
23	10311	10334	F	23	22310	22333
23	12400	12423	F	23	12936	12959
23	15974	15997	F	23	26944	26967
23	15980	16003	F	23	22387	22410
23	25567	25590	F	23	26131	26154
23	534	557	F	23	4193	4216
23	4940	4963	F	23	12694	12717
23	5141	5164	F	23	26537	26560
23	5177	5200	F	23	26908	26931
23	5183	5206	F	23	25725	25748
23	5749	5772	F	23	7245	7268
23	6440	6463	F	23	24925	24948
23	8039	8062	F	23	29563	29586
23	8947	8970	F	23	15661	15684
23	9046	9069	F	23	26781	26804
23	9603	9626	F	23	12525	12548
23	9888	9911	F	23	10072	10095
23	12694	12717	F	23	33377	33400
23	14439	14462	F	23	33689	33712
23	18322	18345	F	23	18328	18351

23	25458	25481	F	23	25971	25994
23	29559	29582	F	23	29758	29781
23	30789	30812	F	23	33343	33366
24	15416	15440	F	24	17289	17313
24	890	914	F	24	17856	17880
24	3111	3135	F	24	13352	13376
24	5180	5204	F	24	5181	5205
24	5757	5781	F	24	8793	8817
24	5970	5994	F	24	29022	29046
24	12255	12279	F	24	12868	12892
24	12435	12459	F	24	29562	29586
24	15167	15191	F	24	27906	27930
24	18857	18881	F	24	18911	18935
24	19587	19611	F	24	22816	22840
24	26838	26862	F	24	33483	33507
25	18325	18350	F	25	18334	18359
25	25722	25747	F	25	29157	29182
26	12114	12114	F	26	12667	12667
26	8612	8612	F	26	14812	14812
26	18884	18884	F	26	18938	18938
28	18841	18869	F	28	18949	18977
28	18845	18873	F	28	18899	18927
28	1785	1813	F	28	4835	4863
29	18325	18354	F	29	18328	18357
33	12371	12404	F	33	13004	13037
35	18834	18869	F	35	18888	18923
35	18911	18946	F	35	18965	19000
38	18899	18937	F	38	18953	18991
39	18834	18873	F	39	18942	18981
39	17158	17197	F	39	30778	30817
42	9809	9851	F	42	33400	33442
46	15340	15386	F	46	17206	17252
46	17206	17252	F	46	30826	30872
52	12335	12387	F	52	12968	13020
53	15333	15386	F	53	17199	17252
53	17199	17252	F	53	30819	30872
53	12342	12395	F	53	12975	13028
60	4963	5023	F	60	9809	9869
63	9788	9851	F	63	33379	33442
70	9781	9851	F	70	33372	33442
73	4942	5015	F	73	9788	9861
74	15312	15386	F	74	17178	17252
75	15312	15387	F	75	30798	30873

76	4937	5013	F	76	33374	33450
78	17174	17252	F	78	30794	30872
83	13470	13553	F	83	14341	14424
87	14039	14126	F	87	19470	19557
87	15300	15387	F	87	30786	30873
22	5129	5151	R	22	5129	5151
22	6980	7002	R	22	15664	15686
22	7086	7108	R	22	22379	22401
22	9122	9144	R	22	16158	16180
22	9431	9453	R	22	9492	9514
22	15971	15993	R	22	26950	26972
22	25524	25546	R	22	25524	25546
22	26950	26972	R	22	26955	26977
22	708	730	R	22	5114	5136
22	1211	1233	R	22	5677	5699
22	1404	1426	R	22	28663	28685
22	1766	1788	R	22	5969	5991
22	3127	3149	R	22	7351	7373
22	4913	4935	R	22	7144	7166
22	5097	5119	R	22	19699	19721
22	5106	5128	R	22	6849	6871
22	9634	9656	R	22	15861	15883
22	9747	9769	R	22	22831	22853
22	10021	10043	R	22	15232	15254
22	10544	10566	R	22	29169	29191
22	11540	11562	R	22	24113	24135
22	12049	12071	R	22	33572	33594
22	13349	13371	R	22	27071	27093
22	14011	14033	R	22	22388	22410
22	14958	14980	R	22	31252	31274
22	15099	15121	R	22	20611	20633
22	15898	15920	R	22	27297	27319
22	15971	15993	R	22	33175	33197
22	23464	23486	R	22	23871	23893
22	23860	23882	R	22	27383	27405
22	26844	26866	R	22	26945	26967
23	24780	24803	R	23	24780	24803
23	14804	14827	R	23	15982	16005
23	32779	32802	R	23	32785	32808
23	714	737	R	23	33170	33193
23	1205	1228	R	23	15972	15995
23	3817	3840	R	23	8277	8300
23	5951	5974	R	23	27781	27804

23	7221	7244	R	23	7228	7251
23	10309	10332	R	23	22916	22939
23	14553	14576	R	23	29556	29579
23	14925	14948	R	23	14932	14955
23	16156	16179	R	23	28932	28955
23	19285	19308	R	23	23262	23285
23	22615	22638	R	23	22621	22644
23	22864	22887	R	23	29831	29854
23	23454	23477	R	23	28280	28303
23	24040	24063	R	23	25391	25414
24	6449	6473	R	24	16200	16224
24	8055	8079	R	24	8065	8089
24	10177	10201	R	24	10181	10205
24	12435	12459	R	24	14969	14993
25	23329	23354	R	25	23329	23354
25	10335	10360	R	25	33078	33103
22	4911	4933	C	22	15756	15778
22	12164	12186	C	22	22525	22547
22	16206	16228	C	22	23416	23438
22	1200	1222	C	22	29690	29712
22	1776	1798	C	22	12528	12550
22	1898	1920	C	22	16223	16245
22	3068	3090	C	22	5050	5072
22	4006	4028	C	22	17002	17024
22	4417	4439	C	22	9096	9118
22	4833	4855	C	22	28285	28307
22	5132	5154	C	22	17064	17086
22	6001	6023	C	22	14516	14538
22	6500	6522	C	22	17305	17327
22	7731	7753	C	22	19569	19591
22	7810	7832	C	22	10280	10302
22	10169	10191	C	22	13618	13640
22	10193	10215	C	22	23190	23212
22	10296	10318	C	22	30655	30677
22	12042	12064	C	22	22719	22741
22	12528	12550	C	22	24983	25005
22	15207	15229	C	22	22383	22405
22	17309	17331	C	22	33692	33714
22	20982	21004	C	22	29785	29807
22	26381	26403	C	22	29169	29191
22	26797	26819	C	22	29237	29259
22	28037	28059	C	22	29181	29203
23	13350	13373	C	23	29746	29769

23	5514	5537	C	23	33681	33704
23	15211	15234	C	23	15971	15994
23	24473	24496	C	23	25111	25134
25	24652	24677	C	25	28150	28175
22	5185	5207	P	22	12531	12553
22	26839	26861	P	22	26907	26929
22	1791	1813	P	22	15431	15453
22	4832	4854	P	22	29816	29838
22	6452	6474	P	22	6452	6474
22	12530	12552	P	22	12530	12552
22	17847	17869	P	22	31399	31421
22	26421	26443	P	22	26421	26443
22	28483	28505	P	22	28486	28508
22	32640	32662	P	22	32640	32662
22	1255	1277	P	22	15656	15678
22	2871	2893	P	22	25723	25745
22	2993	3015	P	22	30649	30671
22	4841	4863	P	22	15431	15453
22	4907	4929	P	22	24768	24790
22	5182	5204	P	22	29167	29189
22	5184	5206	P	22	33222	33244
22	5422	5444	P	22	22664	22686
22	6322	6344	P	22	25392	25414
22	6440	6462	P	22	27990	28012
22	6550	6572	P	22	23854	23876
22	9112	9134	P	22	23750	23772
22	9571	9593	P	22	31780	31802
22	9612	9634	P	22	29160	29182
22	12047	12069	P	22	25680	25702
22	12192	12214	P	22	22714	22736
22	12400	12422	P	22	26671	26693
22	12530	12552	P	22	25728	25750
22	12531	12553	P	22	29163	29185
22	12718	12740	P	22	13362	13384
22	13469	13491	P	22	30138	30160
22	15979	16001	P	22	22464	22486
22	16837	16859	P	22	18157	18179
22	18998	19020	P	22	33292	33314
22	19214	19236	P	22	26871	26893
22	19602	19624	P	22	26060	26082
22	22631	22653	P	22	25721	25743
22	25087	25109	P	22	33297	33319
22	25383	25405	P	22	26950	26972

22	25725	25747	P	22	29167	29189
22	28671	28693	P	22	29687	29709
22	28830	28852	P	22	33492	33514
22	29168	29190	P	22	33800	33822
23	1324	1347	P	23	15967	15990
23	13353	13376	P	23	13355	13378
23	2869	2892	P	23	9751	9774
23	5178	5201	P	23	22631	22654
23	5182	5205	P	23	5189	5212
23	6324	6347	P	23	9914	9937
23	8828	8851	P	23	8828	8851
23	12527	12550	P	23	28487	28510
23	12935	12958	P	23	26671	26694
23	13661	13684	P	23	28752	28775
23	14310	14333	P	23	28663	28686
23	14839	14862	P	23	33702	33725
23	15210	15233	P	23	26950	26973
23	24487	24510	P	23	28658	28681
23	24677	24700	P	23	27092	27115
23	25689	25712	P	23	25689	25712
23	26365	26388	P	23	29746	29769
23	29556	29579	P	23	29556	29579
23	30052	30075	P	23	31524	31547
24	4912	4936	P	24	4912	4936
24	22628	22652	P	24	26912	26936
24	1197	1221	P	24	14475	14499
24	5181	5205	P	24	5188	5212
24	6322	6346	P	24	25765	25789
24	13003	13027	P	24	13008	13032
24	13307	13331	P	24	19274	19298
24	19570	19594	P	24	19582	19606
24	26903	26927	P	24	33162	33186
25	1192	1217	P	25	10312	10337
25	5046	5071	P	25	14953	14978
25	9612	9637	P	25	25720	25745
25	16280	16305	P	25	16280	16305
25	23373	23398	P	25	33695	33720
27	10851	10878	P	27	10851	10878
27	2495	2522	P	27	2495	2522

Forward (direct) show [-F], Reverse show [-R], complement show [-C], Palindromic show [-P]

Supplementary Table S5: Inventory of Tandem Repeat Sequence Identified in the Individual Strians

Species	Indices	Period Size	Copy Number	Consensus Size	Percent Matches	Percent Indels	Score	Entropy (0-2)
P.fici W106-1	5896--5927	15	2.1	15	94	0	55	1.12
P.fici W106-1	17631--17691	23	2.7	23	82	5	79	0.85
P.fici W106-1	17637--17691	12	4.8	11	86	6	74	0.84
P.fici W106-1	26837--26930	45	2.1	45	95	0	170	1.81
P.fici W106-1	26856--26946	45	2	44	85	8	121	1.8
P.fici W106-1	32964--33006	20	2.2	20	95	0	77	1.84
P.fici W106-1	32993--33036	20	2.2	20	91	0	70	1.95
P.fici W106-1	39399--39439	21	2	21	85	0	55	1.56
P.fici W106-1	42106--42158	24	2.2	25	80	16	67	1.31
P.fici W106-1	47993--48048	24	2.3	24	93	0	94	1.76
P.fici W106-1	58816--58858	21	2	21	82	8	52	1.24
P.fici W106-1	58912--58967	24	2.3	24	100	0	112	1.81
P.fici W106-1	58950--58985	12	3	12	100	0	72	1.96
P.fici W106-1	58926--58985	24	2.5	24	86	0	75	1.94
P.fici W106-1	65348--65385	6	6.2	6	87	6	58	0.91
P.fici W106-1	65351--65389	19	2.1	19	100	0	78	1.01
P.fici W106-1	68242--68281	3	13.3	3	89	0	62	1.57
P.fici W106-1	68685--68744	18	3.3	18	95	0	102	1.75
P.fici W106-1	69105--69145	3	14	3	82	14	50	0.95
N.cubana LD08	14938--14978	18	2.3	19	87	8	59	1.36
N.cubana LD08	18326--18368	3	14.3	3	85	0	50	1.68
N.cubana LD08	18759--18793	18	1.9	18	88	0	52	1.76
N.cubana LD08	18835--19000	54	3.1	53	85	5	226	1.84
N.cubana WZS16	8489--8529	18	2.3	19	87	8	59	1.36
N.cubana WZS16	11877--11919	3	14.3	3	85	0	50	1.68
N.cubana WZS16	12310--12344	18	1.9	18	88	0	52	1.76
N.cubana WZS16	12386--12551	54	3.1	53	85	5	226	1.84
N.cubana BT03	13685--13725	18	2.3	19	87	8	59	1.36
N.cubana BT03	17073--17115	3	14.3	3	85	0	50	1.68
N.cubana BT03	17506--17540	18	1.9	18	88	0	52	1.76
N.cubana BT03	17582--17747	54	3.1	53	85	5	226	1.84

Supplementary Table S6: Microsatellite DNA in *N.cubana* LD08,WZS016,BT03 and *P.fici*W106-1

N.cubana LD08					
**SSR nr.**	**SSR type**	**SSR**	**size**	**start**	**end**
1	p1	(T)10	10	1212	1221
2	p1	(A)10	10	1326	1335
6	p1	(A)11	11	5187	5197
8	p1	(T)11	11	6335	6345
9	p1	(T)12	12	7969	7980
11	p1	(T)11	11	12418	12428
12	p1	(T)10	10	12542	12551
13	p1	(A)10	10	14476	14485
17	p1	(A)10	10	19283	19292
19	p1	(A)11	11	25767	25777
20	p1	(A)10	10	26708	26717
4	p3	(TTA)4	12	3507	3518
5	p3	(TAT)5	15	3760	3774
15	p3	(ATG)5	15	18326	18340
22	p3	(TAT)4	12	32059	32070
3	p4	(ATTA)3	12	1757	1768
7	p4	(TTAT)3	12	5967	5978
10	p4	(TATT)3	12	10547	10558
18	p4	(TTTG)3	12	21834	21845
21	p4	(TTTA)3	12	26957	26968
23	p4	(TTTA)3	12	33304	33315
14	p6	(TAGCTA)3	18	14884	14901
16	p6	(TTCATC)3	18	19125	19142
N.cubana WZS16					
**SSR nr.**	**SSR type**	**SSR**	**size**	**start**	**end**
1	p1	(T)10	10	20	29
6	p1	(A)12	12	8025	8036
10	p1	(A)10	10	12834	12843
12	p1	(A)11	11	19318	19328
13	p1	(A)11	11	20259	20269
17	p1	(T)10	10	28611	28620
18	p1	(A)10	10	28725	28734
22	p1	(A)10	10	31332	31341
24	p1	(T)11	11	32479	32489
25	p1	(T)12	12	34113	34124
27	p1	(T)11	11	38562	38572
4	p2	(TA)5	10	2545	2554
8	p3	(ATG)5	15	11877	11891
15	p3	(TAT)4	12	25612	25623

20	p3	(TTA)4	12	29652	29663
21	p3	(TAT)5	15	29905	29919
2	p4	(TGGG)3	12	1194	1205
3	p4	(TTAA)3	12	2094	2105
11	p4	(TTTG)3	12	15385	15396
14	p4	(TTTA)3	12	20510	20521
16	p4	(TTTA)3	12	26857	26868
19	p4	(ATTA)3	12	29156	29167
23	p4	(TTAT)3	12	32111	32122
26	p4	(TATT)3	12	36691	36702
5	p5	(GTATA)3	15	4311	4325
7	p6	(TAGCTA)3	18	8435	8452
9	p6	(TTCATC)3	18	12676	12693
***N.cubana* BT03**					
**SSR nr.**	**SSR type**	**SSR**	**size**	**start**	**end**
1	p1	(T)10	10	1212	1221
2	p1	(A)10	10	1326	1335
6	p1	(A)10	10	3933	3942
8	p1	(T)11	11	5080	5090
9	p1	(T)12	12	6714	6725
11	p1	(T)11	11	11163	11173
12	p1	(T)10	10	11287	11296
13	p1	(G)10	10	11871	11880
14	p1	(A)10	10	13223	13232
18	p1	(A)10	10	18030	18039
20	p1	(A)10	10	24514	24523
21	p1	(A)10	10	25454	25463
4	p3	(TTA)4	12	2253	2264
5	p3	(TAT)5	15	2506	2520
16	p3	(ATG)5	15	17073	17087
23	p3	(TAT)4	12	30806	30817
3	p4	(ATTA)3	12	1757	1768
7	p4	(TTAT)3	12	4712	4723
10	p4	(TATT)3	12	9292	9303
19	p4	(TTTG)3	12	20581	20592
22	p4	(TTTA)3	12	25704	25715
24	p4	(TTTA)3	12	32051	32062
15	p6	(TAGCTA)3	18	13631	13648
17	p6	(TTCATC)3	18	17872	17889
***P.fici* W106-1**					
**SSR nr.**	**SSR type**	**SSR**	**size**	**start**	**end**
2	p1	(T)12	12	2514	2525
4	p1	(A)10	10	2982	2991

5	p1	(T)10	10	3085	3094
7	p1	(T)10	10	5547	5556
8	p1	(T)11	11	6487	6497
9	p1	(A)11	11	6898	6908
10	p1	(A)10	10	7125	7134
12	p1	(A)10	10	7835	7844
13	p1	(A)10	10	11654	11663
17	p1	(A)10	10	25351	25360
18	p1	(T)10	10	25791	25800
19	p1	(A)10	10	25964	25973
20	p1	(C)11	11	28083	28093
24	p1	(A)10	10	32832	32841
27	p1	(A)10	10	38618	38627
30	p1	(T)11	11	42043	42053
32	p1	(A)11	11	43008	43018
33	p1	(A)10	10	46981	46990
35	p1	(T)10	10	52975	52984
36	p1	(T)12	12	54858	54869
37	p1	(A)10	10	59460	59469
38	p1	(T)10	10	59524	59533
39	p1	(T)10	10	59689	59698
41	p1	(A)10	10	62044	62053
42	p1	(T)10	10	62276	62285
43	p1	(A)12	12	62541	62552
46	p1	(A)10	10	65484	65493
50	p1	(T)12	12	69256	69267
26	p2	(AT)5	10	37637	37646
47	p2	(AC)5	10	65519	65528
21	p3	(AAT)4	12	28646	28657
22	p3	(TAT)4	12	29495	29506
28	p3	(TTA)4	12	40554	40565
29	p3	(TAT)5	15	40807	40821
31	p3	(AAT)4	12	42143	42154
48	p3	(ATG)6	18	68242	68259
49	p3	(ATA)6	18	69122	69139
1	p4	(TTTG)3	12	2340	2351
3	p4	(ATTT)3	12	2875	2886
6	p4	(TCTT)3	12	3535	3546
11	p4	(TTTA)4	16	7744	7759
14	p4	(TAAG)3	12	11823	11834
23	p4	(TTCC)3	12	32067	32078
25	p4	(ATTA)3	12	34106	34117
34	p4	(TTAT)3	12	49599	49610

40	p4	(TATT)3	12	60409	60420
16	p5	(ATATG)3	15	15557	15571
15	p6	(AACAGG)3	18	14612	14629
44	p6	(GCTATT)3	18	64770	64787
45	p6	(TAAAAA)3	18	65348	65365

Supplementary Table S7: Taxonomic information and GenBank accession number of the filamentous Ascomycota species used in this study.

Family	Genus	Species	Accession
Erysiphaceae	Erysiphe	quercicola	NC_071210.1
	Erysiphe	pisi	MT880589.1
	Erysiphe	necator	MT880588.1
	Blumeria	graminis	MT880591.1
	Golovinomyces	cichoracearum	MT880590.1
	Podosphaera	xanthii	MK674497.1
Apiosporaceae	Arthrinium	arundinis	NC_035508.1
Arthoniaceae	Arthonia	susa	MH015348.1
Aspergillaceae	Penicillium	citrinum	NC_047444.1
	Penicillium	polonicum	NC_030172.1
	Penicillium	solitum	NC_016187.1
	Penicillium	digitatum	HQ622809.1
	Penicillium	roqueforti	KR952335.1
	Aspergillus	niger	PRJNA15772
	Penicillium	canescens	MW715691.1
Astrosphaeriellaceae	Pithomyces	chartarum	NC_035636.1
Bionectriaceae	Clonostachys	rosea	KU668563.1
	Clonostachys	rogersoniana	MW030499.1
	Clonostachys	compactiuscula	MW030498.1
Botryosphaeriaceae	Macrophomina	phaseolina	MW557546.1
Ceratocystidaceae	Ceratocystis	papillata	MT331844.1
	Ceratocystis	fimbriata	MT331841.1
	Ceratocystis	colombiana	MT331839.1
	Ceratocystis	lukuohia	NC_056158.1
	Ceratocystis	albifundus	MG976800.1
	Ceratocystis	polychroma	MT331817.1
	Ceratocystis	cacaofunesta	NC_020430.1
	Ceratocystis	uchidae	MT331819.1
	Ceratocystis	changhui	MT331818.1
	Ceratocystis	huliohia	NC_056157.1
	Endoconidiophora	resinifera	MK012641.1
Cladoniaceae	Cladonia	petrophila	MG941021.1
	Cladonia	subtenuis	MG949117.1
	Cladonia	macilenta	MK318967.1
	Cladonia	rangiferina	KY460674.1
	Cladonia	peziziformis	MG686615.1
	Cladonia	apodocarpa	NC_039372.1
Cladosporiaceae	Cladosporium	sphaerospermum	MN657181.1
	Cladosporium	anthropophilum	NC_061970.1
	Cladosporium	cladosporioides	MN661341.1
Clavicipitaceae	Epichloe	hybrida	KX066187.1

	Epichloe	festucae	KX066186.1
	Epichloe	typhina	KX066185.1
	Paecilomyces	penicillatus	MK069583.1
	Pochonia	chlamydosporia	NC_022835.1
	Metarhizium	anisopliae	NC_008068.1
	Orbiocrella	petchii	MT447058.1
Coccocarpiaceae	Coccocarpia	palmicola	KY362513.1
Coniothyriaceae	Coniothyrium	glycines	MH337273.1
Cordycipitaceae	Cordyceps	chanhua	MH734937.2
	Lecanicillium	saksenae	NC_028330.1
Cryphonectriaceae	Cryphonectria	parasitica	KT428651.1
	Chrysoporthe	austroafricana	NC_030522.1
	Chrysoporthe	cubensis	KT380885.1
	Chrysoporthe	deuterocubensis	KT380884.1
Diaporthaceae	Diaporthe	nobilis	MT982436.1
	Diaporthe	longicolla	MT527962.1
Didymellaceae	Didymella	pinodes	KT946597.1
Dothioraceae	Sydowia	polyspora	MK637641.1
Drepanopezizaceae	Marssonina	brunnea	NC_015991.1
Glomerellaceae	Colletotrichum	acutatum	KR349346.1
	Colletotrichum	fioriniae	KU375885.1
	Colletotrichum	lindemuthianum	KF953885.1
	Colletotrichum	gloeosporioides	KX885104.1
	Colletotrichum	aenigma	KX885105.1
	Colletotrichum	fructicola	KX034082.1
	Colletotrichum	siamense	MW929093.1
	Colletotrichum	lupini	KT918406.1
	Colletotrichum	salicis	KY774449.1
	Colletotrichum	tamarilloi	KU196965.1
Graphidaceae	Gomphillus	americanus	KY353115.1
	Graphis	lineola	KY315996.1
Helotiales	Cairneyella	variabilis	NC_029759.1
Hypocreaceae	Trichoderma	reesei	NC_003388.1
	Trichoderma	lixii	NC_052832.1
	Trichoderma	cornu	MW525445.1
	Trichoderma	harzianum	MN564945.1
	Trichoderma	gamsii	NC_030218.1
	Trichoderma	asperellum	NC_037075.1
	Trichoderma	atroviride	NC_048477.1
Hypoxylaceae	Annulohypoxylon	stygium	MH620794.1
Lecanoraceae	Lecanora	saxigena	NC_042183.1
	Lecanora	strobilina	NC_030051.1
	Lecanora	cinereofusca	NC_042184.1
Mollisiaceae	Phialocephala	subalpina	NC_015789.1

Mycosphaerellaceae	Pseudocercospora	mori	NC_037198.1
	Pseudocercospora	fijiensis	NC_044132.1
	Cercospora	nicotianae	OK075294.1
	Zymoseptoria	graminicola	NC_010222.1
	Zymoseptoria	tritici	MH374028.1
Nectriaceae	Fusarium	solani	NC_016680.1
	Fusarium	anthophilum	MT010928.1
	Fusarium	andiyazi	MT010917.1
	Fusarium	verticillioides	MT010915.1
	Fusarium	begoniae	MT010929.1
	Fusarium	napiforme	MT010918.1
	Fusarium	circinatum	JX910419.1
	Fusarium	nygamai	MT010926.1
	Fusarium	bactridioides	MT010927.1
	Fusarium	pseudonygamai	MT010925.1
	Fusarium	guttiforme	MT010931.1
	Fusarium	redolens	MT010909.1
	Fusarium	pseudoanthophilum	MT010924.1
	Fusarium	musae	MT010916.1
	Fusarium	acutatum	MT010914.1
	Fusarium	concentricum	MT010911.1
	Fusarium	brevicatenulatum	MT010923.1
	Fusarium	bambusae	MH684411.1
	Fusarium	globosum	MT010913.1
	Fusarium	foetens	MT010932.1
	Fusarium	pseudocircinatum	MT010920.1
	Fusarium	tricinctum	MT269798.1
	Fusarium	commune	NC_036106.1
	Fusarium	ananatum	MT010930.1
	Fusarium	denticulatum	MT010934.1
	Fusarium	annulatum	MT010912.1
	Fusarium	ficicrescens	MT010922.1
	Fusarium	lactis	MT010921.1
	Fusarium	ramigenum	MT010919.1
	Fusarium	oxysporum	NC_017930.1
	Fusarium	pseudograminearum	MT036635.1
	Fusarium	cerealis	MT036639.1
	Fusarium	mangiferae	KP742838.1
	Fusarium	asiaticum	MN935352.1
	Fusarium	gerlachii	KM486533.1
	Fusarium	graminearum	DQ364632.1
	Fusarium	culmorum	KP827647.1
	Calonectria	ilicicola	NC_046826.1
	Nectria	cinnabarina	NC_030252.1

	Ilyonectria	destructans	NC_030340.1
Onygenales	Paracoccidioides	brasiliensis	AY955840.1
Opegraphaceae	Opegrapha	vulgata	NC_035825.1
Ophiocordycipitaceae	Ophiocordyceps	sinensis	NC_034659.1
Ophiostomataceae	Ophiostoma	piliferum	MW122508.1
	Ophiostoma	novo	MG020143.1
Parmeliaceae	Bryoria	tenuis	NC_034786.1
	Hypogymnia	vittata	NC_035730.1
	Hypomyces	aurantius	KU666552.1
	Pseudevernia	consocians	NC_039163.1
	Usnea	ceratina	KX987159.1
Physciaceae	Heterodermia	casarettiana	MH359411.1
	Heterodermia	speciosa	MG711806.1
Plectosphaerellaceae	Verticillium	dahliae	DQ351941.1
	Verticillium	nonalfalfae	NC_029238.1
Pleosporaceae	Bipolaris	oryzae	NC_057095.1
	Bipolaris	sorokiniana	NC_047242.1
	Stemphylium	lycopersici	NC_036039.1
	Bipolaris	cookei	MF784482.1
Ploettnerulaceae	Rhynchosporium	secalis	NC_023128.1
	Rhynchosporium	commune	NC_023126.1
	Rhynchosporium	agropyri	NC_023125.1
Podosporaceae	Podospora	anserina	NC_001329.3
Pseudeurotiaceae	Pseudogymnoascus	destructans	NC_033907.1
	Pseudogymnoascus	pannorum	KR055655.1
Ramalinaceae	Phyllopsora	corallina	NC_034779.1
Sclerotiniaceae	Botrytis	cinerea	KC832409.1
	Monilinia	laxa	NC_051483.1
	Monilinia	fructicola	MT005827.1
	Sclerotinia	sclerotiorum	NC_035155.1
	Sclerotinia	borealis	NC_025200.1
Sordariaceae	Neurospora	crassa	KY498478.1
Sporocadaceae	Pestalotiopsis	fici	KX870077.1
	Neopestalotiopsis	cubana (WZS016)	NC_071220.1
	Neopestalotiopsis	cubana (BT03)	OQ707026.1
	Neopestalotiopsis	cubana (LD08)	OQ707025.1
Xylariaceae	Nemania	diffusa	NC_049077.1
Hypocreales	Hapsidospora	chrysogena	NC_023268.1
Peronosporaceae	Phytophthora	infestans	MH286885.1
